# GenomeTester4: a toolkit for performing basic set operations - union, intersection and complement on k-mer lists

**DOI:** 10.1186/s13742-015-0097-y

**Published:** 2015-12-03

**Authors:** Lauris Kaplinski, Maarja Lepamets, Maido Remm

**Affiliations:** 1 Department of Bioinformatics, University of Tartu, Riia 23, Tartu, 51010 Estonia; 2 Estonian Biocentre, Riia 23B, Tartu, 51010 Estonia

**Keywords:** K-mers, Sequence analysis, Next-generation sequencing

## Abstract

**Background:**

K-mer-based methods of genome analysis have attracted great interest because they do not require genome assembly and can be performed directly on sequencing reads. Many analysis tasks require one to compare k-mer lists from different sequences to find words that are either unique to a specific sequence or common to many sequences. However, no stand-alone k-mer analysis tool currently allows one to perform these algebraic set operations.

**Findings:**

We have developed the GenomeTester4 toolkit, which contains a novel tool GListCompare for performing union, intersection and complement (difference) set operations on k-mer lists. We provide examples of how these general operations can be combined to solve a variety of biological analysis tasks.

**Conclusions:**

GenomeTester4 can be used to simplify k-mer list manipulation for many biological analysis tasks.

**Electronic supplementary material:**

The online version of this article (doi:10.1186/s13742-015-0097-y) contains supplementary material, which is available to authorized users.

## Findings

### Background

Because of the rapid uptake and progress of next-generation sequencing techniques, both the time and cost required to sequence full or partial genomes has decreased dramatically. On the other hand, these new technologies have introduced new bioinformatic problems resulting from short read lengths, a large number of sequencing errors, and a huge amount of data that must be processed. Analysis of genomic data often requires either *de novo* assembly of the genome, mapping of the data to a reference genome, or homology searches from raw reads, all of which are time-consuming and can introduce additional errors.

In recent years, oligomer-frequency-based methods of genome analysis have attracted great interest because they do not require genome assembly and can be performed directly on sequencing reads [1, 2]. These methods have the potential to be both faster and less error-prone than traditional methods, yet have also proven to be useful for correcting sequencing errors during the initial step of mapping and assembly pipelines [3] and to detect overlapping reads from sequencing datasets [4]. Oligomer-frequency-based methods should now be considered general tools for genomic analysis.

Oligomer frequency analysis is typically conducted by k-mers (oligomers of length k). The first step involves counting k-mers from raw sequencing reads or assembled sequences and is performed in an analogous manner for all subsequent k-mer analysis methods. Several k-mer counting programs have been developed in recent years, both as part of assembly tools or as separate programs. One of the fastest and most widely used k-mer counting tools is Jellyfish [5], which runs on several parallel CPU threads and operates on a lock-free hash table that eliminates waiting for concurrent data access from different threads. In addition to hashing, k-mer counters can also use more complex data structures that facilitate optimal counting for specific cases. For example, Tallymer [6] uses a suffix array and specializes in counting k-mers from large eukaryotic genomes with many repeated sequences. KMC2 [7] and DSK [8] can run on computers with limited memory by writing k-mers into several small temporary tables that are combined onto disk storage. Turtle [9] uses a combination of Bloom filter and sort-and-compact to reduce cache misses while operating on large datasets.

The second step - k-mer analysis - is task-specific. Analysis of k-mer frequencies has been used to estimate the size of the genome [3], to detect de novo repeats [6], to measure gene expression [2], to find similar reads from different metagenomic samples [10] and to identify bacteria from sequencing reads using k-mer distributions or specific marker sequences [1]. GSMer [4] can be used to find taxon-specific barcodes using k-mer counting, whereas Kraken [1] classifies bacteria by first adding phylogenetic information to every k-mer in its database and then estimating the origin of the bacteria by its k-mer content. The Khmer package implements many common k-mer operations for short-read sequencing [11].

Many k-mer analysis tasks would benefit from the use of mathematical set operations to find sub- and supersets of k-mers from different nucleotide sequences. Here we present the GenomeTester4 toolkit that aids in both the creation and the modification of k-mer lists. GenomeTester4 can generate lists of k-mer counts from nucleotide sequences and perform basic algebraic set operations - union, intersection and difference (complement) - on these lists. Currently there is no other public toolkit that provides this functionality. We describe the toolkit and provide examples of how to apply this functionality to perform specific biological analyses.

### Overview and working principle of the GenomeTester4 toolkit

**Fig. 1:**
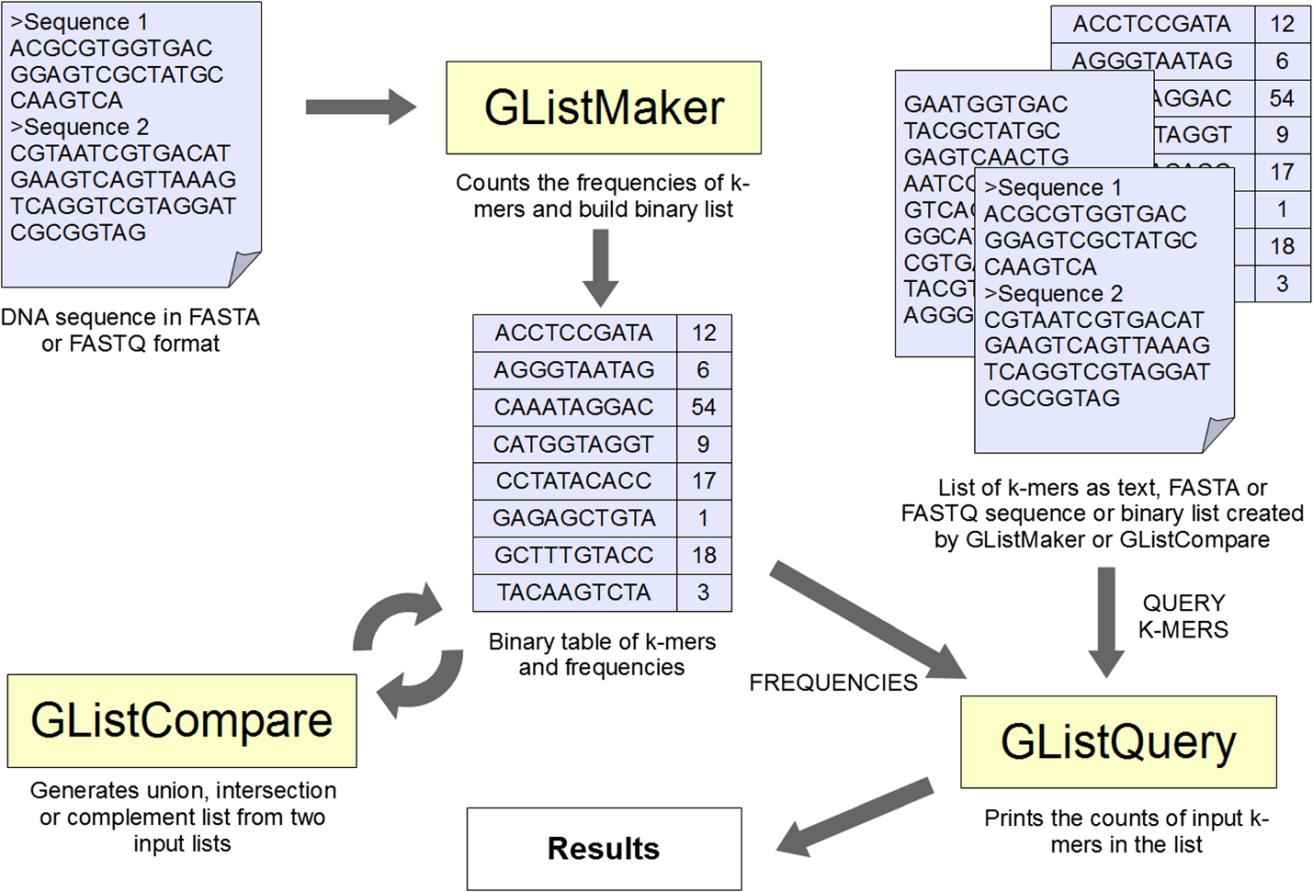
A schematic overview of the workflow of the programs in the GenomeTester4 package. GListMaker takes FASTA or FASTQ as input and builds a binary list of k-mer counts. GListCompare performs set operations with two k-mer lists and generates a new list as output. GListQuery can be used to look up the counts from a list using either a text file, FASTA/FASTQ file or another k-mer list as input

The GenomeTester4 software package consists of three programs - GListMaker, GListCompare and GListQuery (Fig. 1). The GListMaker routine generates k-mer count lists from nucleotide sequences, and the GListCompare tool performs basic algebraic set operations with these lists. The GListQuery tool searches for user-provided sequences from lists generated either by GListMaker or GListCompare. Together, these tools can be used to construct custom k-mer analysis pipelines for a variety of applications. GenomeTester4 is written in the C programming language and can be run from the command line on Linux or other Unix-like operation systems.

#### K-mer list format

The central data structure of all three programs is a binary k-mer list that consists of a small header and an array of k-mers together with their counts. K-mers are encoded as 64-bit unsigned integers with two bits representing each nucleotide. Counts are 32-bit unsigned integers. To reduce memory usage, the list contains only the canonical form of each k-mer. This means that one data entry represents both a k-mer and its reverse complement and is stored using the smaller of the two possible integer encodings. The maximum length of k-mers is thus 32 nucleotides and the maximum possible total count of any given k-mer and its reverse complement is 2^32^ - 1. The full specification of the k-mer list format is given in Additional file 1.

#### GListMaker

GListMaker computes k-mer counts from nucleotide sequences stored in FASTA or FASTQ format. It uses temporary arrays to collect all k-mers from the input file during the reading phase. The arrays are then sorted and the adjacent instances of the same k-mer are counted during the collation phase. Multiple CPU threads can be used if there is more than one file of input sequences.

#### GListCompare

**Fig. 2:**
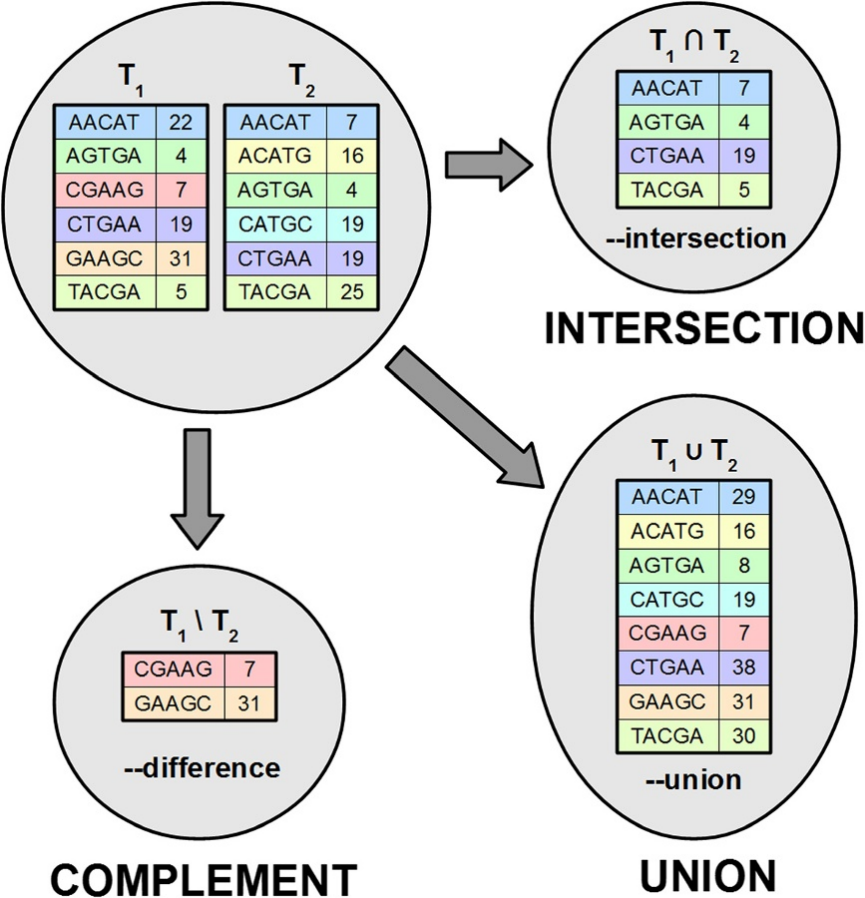
The basic set operations implemented by GListCompare. The default k-mer counts stored in the derived list are the following: for union, the sum of k-mer counts from both lists; for intersection, the smaller of the k-mer counts in either of the lists; and for complement, the k-mer count in the first set. The GListCompare program argument for given set operation s shown below each calculated set

GListCompare performs the basic set-algebraic operations - union, intersection and difference - with two user-specified k-mer lists (Fig. 2). All operations are performed by simultaneously iterating over both lists with O(N) complexity (the working time is proportional to the input list sizes). The resulting new list is streamed directly to disk, thereby requiring very little memory.

The union of two lists contains all entries that were present in either of the original lists, intersection contains only the entries that were present in both lists, and complement provides the entries that were present in the first but not in the second list. The precise definition of what counts as the presence of k-mer and what will be the final count in the output list can be specified by rules. By default, applying a union operation results in a list in which the final k-mer count is the sum of the counts from both initial lists, whereas calculating the intersection results in a default list where the smaller of the two counts is reported. Calculating the complement provides the count from the first list by default (Fig. 2).

**Fig. 3:**
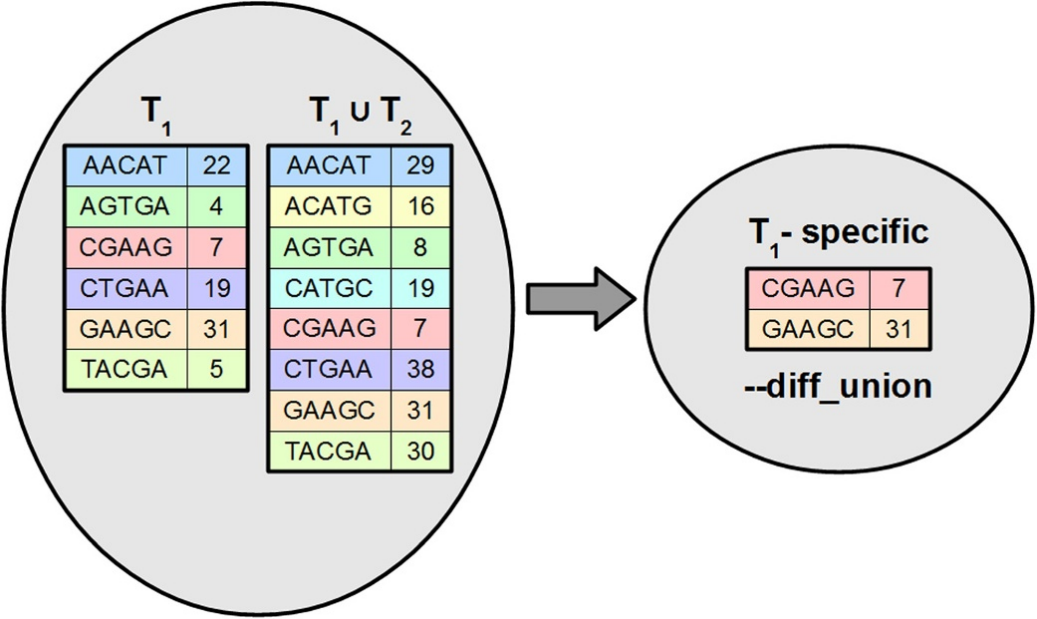
The complement from union function within GListCompare. First, a union of two or more lists is calculated that includes the sample-specific list T_1_. In this example, this list is T_1_ ∪ T_2_. Typically, this union will include k-mers from many species or strains. Next, we find the intersection between T_1_ and this composite union. We define this as the intersection that only includes k-mers that have the same count in both lists. The result is a list of k-mers that are unique to T_1_. Note that in this case the resulting list is the same as that calculated using the complement function in the example given in Fig. 2

To speed up the generation of lists of sample-specific k-mers from a collection of many genomes we have also implemented a function to find what we term the complement from union (Fig. 3). In this case the difference is calculated with the assumption that the second list is a union containing the first list. Whenever the counts of some k-mer in both lists are equal it means that the first list is the only one among lists in union containing this k-mer and thus it is included in the output list. This function is useful because the same composite list can be used to find the specific k-mers for each of the samples used to compose it, thus eliminating the need to calculate separate control lists for each test of uniqueness. GListCompare also supports finding a complement with a user-specified number of mismatches. In this case the complement outputs the k-mers that are present in the first list but differ from any k-mer in the second list by at least m positions. This is calculated in a step-wise process where at first the complement with no mismatches is calculated, then the retrieved k-mers are searched with one mismatch, then two mismatches and so on, up until the user-specified number of mismatches. Only the k-mers that are not found from the second list with the previous number of mismatches will be considered in the next step. At every step, the relevant k-mers are stored in an array in RAM.

#### GListQuery

GListQuery is used to query the counts of k-mers from k-mer lists. The input can be single k-mer sequences from the command line, lists of k-mers supplied in a text file, or other k-mer lists generated with GListMaker or GListCompare. Input k-mers can also be read from FASTA/FASTQ files with a sliding window. K-mers are looked up by binary search, resulting in O(logN) complexity (the working time is proportional to the logarithm of input list size).

GListQuery can also search for k-mers that differ from the query k-mer by up to a user-supplied number of mismatches. To accomplish this, it generates all possible mismatched k-mer versions of each query sequence and searches for each of them from the input list.

#### Optimizations

GListCompare and GListQuery do not read lists directly into RAM but instead memory-map these using the Linux mmap system function. This guarantees that only those parts of each file that are being used will be read from disk to RAM. Parts that are never used will not be read and those that were not used recently will be silently cleaned from memory without virtual memory swapping. This allows the system to use lists larger than the available amount of physical memory, albeit with a performance penalty if many look-ups are performed sequentially.

GListMaker uses a pool of CPU worker threads to speed up list generation. Each input file is processed in parallel if enough worker threads are available. The sorting and merging of temporary tables into the final k-mer list is also performed using separate worker threads.

#### The rules of set operations

To perform the basic set-algebraic operations of union, intersection and difference on k-mer lists, we made additional design decisions. The semantics of standard mathematical set operations are defined only by the presence or absence of specific elements in the set. In case of k-mer lists we have richer structure, as for each k-mer there is also the count of its occurrences.

To accommodate the occurrence count within k-mer lists, we extended these operations by defining the semantics of presence or absence together with a method to calculate the final count. For all operations, the k-mer is counted as being present in one of the input lists only if its count is equal to or above a minimum cutoff value. After determining the presence in the two individual lists, the standard set operation is then applied. If by the result of the operation the k-mer should be included in the output set, its final count is calculated by predetermined rule and the k-mer is written to the output list.

The possible rules are:

- *1* - set count value to 1
- *2* - set count value to 2
- *add* - add up both counts
- *subtract* - subtract the second count from the first
- *min* - smaller of the counts
- *max* - larger of the counts
- *first* - count in the first list
- *second* - count in the second list

The default rule for intersection is *min*, for union *add* and for difference *first*.

To avoid the possibility of zero valued counts in the output list, union and difference operations implement a subset of these rules.

### Data and resources used in examples

All bacterial genomes used were downloaded from the RefSeq microbial genomes ftp site [12] and were current as of 18 June 2014. Plasmids and sequences smaller than 0.5 Mbp (million base pairs) were excluded from the dataset.

Human genome build 37.1 was downloaded from the RefSeq genomes ftp site [12].

*S. aureus* and human chromosome 14 sequencing read datasets were downloaded from GAGE project [13].

*Bos taurus* genome build 6.1 were downloaded from RefSeq genomes ftp site [12].

### Performance

All software performance tests were conducted on a CentOS 5.10 Linux server with 32 cores (2.27 GHz) and 512 GiB (gibibyte, 2^30^ bytes) RAM.

By design, GListMaker rapidly processes genomic sequences that contain few repeats because it performs counting by sorting and all data is stored in a single list structure. However, it is not well optimized for counting large datasets of next-generation sequencing reads, which contain many repeated k-mers. Because all k-mers from a single input file are normally read into memory during the first step, very large input sequences may easily consume all available memory even if the number of unique k-mers in the input is small. This can be mitigated by using command-line arguments that specify the size of in-memory tables. Each k-mer requires 8 bytes of memory (single 64 bit integer) in the initial reading phase and each unique k-mer requires 12 bytes (one 64-bit and one 32-bit integer) during the collation phase. As GListMaker does not implement Bloom filter and keeps all k-mers are in memory, singleton sequencing errors consume both memory and disk space. For a more detailed analysis of memory usage, please refer to Additional file 2.

The running speed and memory requirements of GListMaker depend on both the total number of k-mers in the input files and the number of unique k-mers. We found that the performance of GListMaker is comparable to that of other k-mer counting tools. Tables 1 and 2 provide a comparison with Jellyfish, KMC and DSK for specific input sequences.

**Table 1:** Comparison of the 32-mer counting speeds of GenomeTester4, Jellyfish 2.2.0, KMC 2.2 and DSK 2.0.7 with a single thread and 24 threads

		*E. coli* K-12 strain MG1655 genome	*H. sapiens* genome	*S. aureus* sequencing reads (GAGE library 1)	*H. sapiens* chromosome 14 sequencing reads (GAGE library 1)
File sizes		1 file, 4.7 Mbp	24 files, 3.0 Gbp	2 files, 86 Mbp	2 files, 2.55 Gbp
1 thread	GListMaker	0.9	978.47	16.61	764.17
	JellyFish	3.64	1923.99	43.1	1222.48
	KMC	0.76	354.32	11.22	338.52
	DSK	2.52	102.41	14.41	361.50
24 threads	GListMaker	1.52	243.44	12.15	261.7
	JellyFish	0.54	112.52	5.65	100.84
	KMC	0.25	41.47	2.48	57.19
	DSK	2.63	60.76	3.51	59.16

All measurements are taken as the mean of five runs and presented in seconds. For KMC, the RAM-only version was used to speed up the counting; for DSK the RAM limit was 200 GiB

**Table 2:** Comparison of the peak memory consumption of GenomeTester4, Jellyfish 2.2.0, KMC 2.2 and DSK 2.0.7 while counting 32-mers with 24 threads

Source sequence	*H. sapiens* genome	*H. sapiens* chromosome 14 sequencing reads (GAGE library 1)
File sizes	24 files, 3.0 Gbp	2 files, 2.55 Gbp
GListMaker	64 GiB	24 GiB
JellyFish	23 GiB	9 GiB
KMC	11 GiB	11 GiB
DSK	32 GiB	2.7 GiB

For KMC, the RAM-only version was used to speed up the counting

The speed of GListQuery depends on the size of the list file being searched. Using a list of 32-mers from a single bacterial genome we obtained a speed of up to 35 million look-ups per minute. Using a 64 GiB union of all NCBI bacterial genomes, the look-up speed was roughly 2.7 million 32-mers per minute.

Because the list files are memory-mapped, more parts of the file will be read into RAM as the number of query sequences increases, which also speeds up subsequent look-ups. This guarantees a near-immediate start-up for the case where only a few look-ups are required because the entire file does not need to be read from disk, while also providing near-optimal speeds for a large number of look-ups.

### Usage examples

We have found that many k-mer analysis tasks require one to combine or partition k-mer lists into sub- and supersets of k-mers. By implementing optimized set-algebraic operations within a single tool, GenomeTester4 allows researchers to significantly simplify k-mer analysis pipelines. Still, because most research questions cannot be implemented using only simple set operations on k-mer lists, we expect that both the initial and final steps of analysis will be performed using custom task-specific tools.

#### Example 1: Counting k-mers in a large set of sequences

If the input sequence is composed of many smaller files, one can combine GListMaker and GListCompare to generate the final list by trading less RAM usage for longer list generation time. For example, we used the following approach to generate the union of all k-mers in NCBI bacterial genome database (2776 whole genomes):

1. Separate k-mer lists were created for each bacterial genome with GListMaker.
2. These lists were recursively combined pairwise with a script MakeUnion.pl (included in GenomeTester4 package) that uses the GListCompare union function.

The running time of this operation is given in Table 3. The maximum amount of memory required was determined by the size of largest bacterial genome.

**Table 3:** GListCompare running times for the generation of different bacterial datasets

List	List size	Number of unique k-mers	Generation time
32-mer lists of all bacteria			2 h 8 m
Union list of all bacteria	64 GiB	5,676,675,273	2 h 30 m
*Streptococcus* genus specific	1.4 KiB	115	75.2 s
*S. pneumoniae* species specific	4 MiB	348,970	79.2 s
*S. pneumoniae* strain G54 specific	922 KiB	78,667	77.3 s

#### Example 2: Finding chromosome-specific repeats in a eukaryotic genome

There are many specific tools that have been developed to find *de novo* repeats; however, none of these allows the user to use control sequences to find repeats that are specific to certain sequences. In general, we expect that repeats in regions with significant homology share a set of common k-mers. We further expect that most of these k-mers are not present elsewhere in significant numbers. To demonstrate how the set operations implemented in GenomeTester4 can be used to accomplish this task, we present an example of how to find sex-chromosome-specific repeats in a cow genome for fluorescence in situ hybridization. The following procedure was used to restrict the search space by finding a specific set of repeated k-mers:

1. Created separate 16-mer lists from X and Y chromosomes with GListMaker
2. Created a single 16-mer list from all autosomes
3. Created subsets of all k-mers whose count was at least 10 in the sex-chromosome lists using the GListCompare cutoff option
4. Subtracted the autosome list from repeated k-mer lists with GListCompare using the difference function with a cutoff value of 2
5. Subtracted the other sex-chromosome list from the repeated and unique k-mer lists using GListCompare with a cutoff value of 1

The resulting lists contained 112,387 Y-specific k-mers. We removed those that had more than 50 copies so as to ignore well-known repeats by:

6. Creating a subset of all k-mers that occur at least 50 times in the chromosome Y list
7. Subtracting the list of k-mers of over 50 copies from the list of Y-specific k-mers using GListCompare difference function

In total, we identified 4878 X-specific and 137,868 Y-specific 16-mers.

The final steps in our analysis were performed using custom Perl scripts. First, we located the regions in chromosome that had significant over-representation of unique repeated k-mers. Then we grouped these regions by similarity using BLAST alignment. Finally, these regions were aligned against the full genome with BLAST. The more complete protocol with command used is outlined in Additional file 3.

#### Example 3: Finding group-specific k-mers to identify bacteria

The detection of bacteria at various phylogenetic levels is often required during medical diagnosis and in both epidemiological and ecological studies. To identify whether a certain bacterium belongs or is closely related to a predefined group of strains, one can find the k-mers that are unique to that group of strains and search for those from sequencing reads of the bacterium of interest. We used GenomeTester4 to generate lists of specific 32-mers from the genus *Streptococcus*, of *S. pneumoniae* species and of *S. pneumoniae* strain G54.

First, we created a union of all k-mers contained in all bacteria in the NCBI database, as described above. During this step we also obtained k-mer lists from all strains of interest.

Next, we found the intersection of all lists of the strains of interest. This list contains all k-mers that are present in all bacteria from the set. It is reasonable to expect that many of these k-mers will be present in any new strain as long as it is closely related to any known strain from this set. Because we want to use the difference from union operation for finding unique k-mers, the intersection operation must use the -sum rule (i.e. the counts in the intersection list are sum of the counts of source lists). Finally, we found the difference between the target list and the list of all bacterial genomes using the difference from union option of GListCompare.

The running times of GListMaker or GListCompare and the sizes of all lists used in this example are provided in Table 3. Although we used assembled genomes to generate target and non-target lists, the assembly process is probably not required for creating lists. One can compile these lists directly from sequencing reads to avoid the time-consuming process of assembly. Also, we expect that a much lower sequencing coverage is required to compile representative k-mer lists than the coverage required for genome assembly. The speed of the GenomeTester4 package allows one to perform this kind of analysis as a routine part of sequencing.

## Conclusions

GenomeTester4 is a universal toolbox for creating and using k-mer lists from genomic sequences and its computational speed is competitive with other k-mer counting programs. This package is unique in its ability to perform fast set operations and list queries with a user-specified number of mismatches. These routines have proven to be useful in our research and, because of their universal nature, we expect that others may find them to be useful for many possible tasks that require k-mer counting and/or operations with k-mer lists. This makes GenomeTester4 a potentially valuable addition to many k-mer and sequence analysis toolkits.

## Availability and requirements

Project name: GenomeTester4

Project (source code) home page: https://github.com/bioinfo-ut/GenomeTester4

Operating systems: Linux (64-bit)

Programming language: C

Other requirements (when recompiling): GCC version 4

License: GNU General Public License version 3.0 (GPLv3)

Any restrictions to use by non-academics: none

## Availability of supporting data

The data sets supporting the results of this article are available in the GigaDB repository [14].

## Additional files

Additional file 1: **The GenomeTester4 file format.** (PDF 48 kb) Additional file 2: **Memory and CPU usage.** (PDF 68 kb) Additional file 3: **Protocol.** (PDF 24 kb)

## Abbreviations

Gbp: 1 billion base pairs
GiB: gibibyte (2^30^ bytes)
KiB: kibibyte (2^10^ bytes)
Mbp: 1 million base pairs
MiB: mebibyte (2^20^ bytes)
