# Supplementary material for: GenomeTester4: a toolkit for performing basic set operations - union, intersection and complement on k-mer lists
Source: Gigascience. 2015 Dec 3;4:58. doi: 10.1186/s13742-015-0097-y (PMC4669650; doi:10.1186/s13742-015-0097-y)
Supplement: Additional file 1: — The GenomeTester4 file format. (PDF 48 kb) [file 13742_2015_97_MOESM1_ESM.pdf]

GenomeTester4 file format

GenomeTester4 k-mer list is binary file, encoded in little-endian (x86 default) byte order. It is composed of header and table parts.

Header (40 bytes)

datatype	bytes	name	description
byte[4]	4	code	'C','4','T','G', used as a “magic” tag to identify GenomeTester list files.
int32	4	major version	used to check the compatibility of the list with GenomeTester program
int32	4	minor version	used to check the compatibility of the list with GenomeTester program
int32	4	word length	k-mer length
int64	8	number of words	the number of unique k-mer entries in the table part of file
int64	8	number of k-mers	the total number of k-mers in sequence
byte[8]	8	padding	reserved for future versions

The table section follows immediately after the end of header. The table is composed of interleaved k-mers (encoded as 64 bit integer) and their counts (stored in 32 bit integer). Each k-mer entry thus uses 12 bytes in the table.

K-mers are encoded with the following bit pattern:
A 00
C 01
G 10
T 11
The 3' end of k-mer is always stored in least significant two bits (0-1) of the integer. If the length of k-mer is less than 32 bp, the unused significant bits are filled with zeros.
