# Supplementary material for: GenomeTester4: a toolkit for performing basic set operations - union, intersection and complement on k-mer lists
Source: Gigascience. 2015 Dec 3;4:58. doi: 10.1186/s13742-015-0097-y (PMC4669650; doi:10.1186/s13742-015-0097-y)
Supplement: Additional file 2: — Memory and CPU usage. (PDF 68 kb) [file 13742_2015_97_MOESM2_ESM.pdf]

Memory and CPU usage

According to our analysis, reading the input files and the final merging steps are the most time-consuming processes. Thus, the optimal number of threads is one or a few more than there are input files.

By default, GListMaker reads all k-mers from one input file that is less than 500Mbp long into a temporary table that will subsequently be sorted and counted. The amount of memory required per file is:

8 * N_kmers_in_file + 12 * N_unique_kmers_in_file

The total amount of memory needed for the final list is:

12 * N_unique_kmers

Because one temporary table is reused for the construction of the final list, the required memory is slightly less than the sum of the sizes of temporary tables and the final list.

If memory is limited, the number and size of temporary tables can be constrained with a penalty on processing time. In such cases, GListMaker parses the input files into blocks of a specified size. Those blocks are then sorted, counted, and merged into the final list. The maximum amount of memory required in this case is:

12 * N_unique_kmers + N_temporary_tables * 12 * average_table_size

Initially, all tables are the same size as the input blocks. Because tables are merged pairwise, the size of some tables grows. One exception is the case when two temporary tables are specified. In this case one table grows and the other remains the same.

We have found that the average amount of memory required for counting multiple files with a block size smaller than the file size is:

24 * N_unique_kmers

At least one temporary table is required. Also, because each task (reading sequence, sorting table, and merging tables) requires one or two tables to work on, it is not possible for the program to use more threads than the number of temporary tables.

Possible areas of improvement

Generating large lists consumes a considerable amount of memory. It is possible to limit the amount of RAM that will be used for temporary buffers, however, this lowers the list generation speed. On memory-constrained systems one can also process all input files individually into separate list files and then merge these into a final list with GlistCompare. This approach has comparable speed but uses very little memory.

The O(logN) binary search is a potential place of improvement for GListQuery. One potential way to increase the performance is to load k-mers into a radix tree, which would allow lookups in linear time. Still, this approach has certain drawbacks. If the tree structure is stored in a file together with the list, streamed set operations are not possible. If, on the other hand, the tree is constructed during run-time, it requires parsing the entire list into memory instead of using memory-mapping. One potential way to avoid both problems is to construct the tree dynamically so that if a word is not found in the tree it will be looked up by a binary search and then the relevant nodes can be created. Another way would be to create a small lookup table and use a hybrid approach so that only the suffix of a k-mer is looked up by binary search.
