# Supplementary material for: GenomeTester4: a toolkit for performing basic set operations - union, intersection and complement on k-mer lists
Source: Gigascience. 2015 Dec 3;4:58. doi: 10.1186/s13742-015-0097-y (PMC4669650; doi:10.1186/s13742-015-0097-y)
Supplement: Additional file 3: — Protocol. (PDF 24 kb) [file 13742_2015_97_MOESM3_ESM.pdf]

Protocol

Locating novel X and Y chromosome specific repeats in cow genome (Bos taurus 4.6.1).

We expected that repeated regions have at least some common 16-mers and these 16-mers are not present in other chromosomes in significant numbers. We used cutoff 2 (i.e. at most single occurrence) for comparing autosomes and cutoff 1 (i.e. not a single occurrence) for comparing with other sex chromsome.

1. Created separate 16-mer lists X and Y chromosomes
glistmaker chrX.fa -w 16
Running time: 0m29.400s
NUnique 55340218
NTotal 83004526
glistmaker chrY.fa -w 16
Running time: 0m11.759s
NUnique 3869143
NTotal 38719749

2. Created single 16-mer list for all autosomes
glistmaker autosomes.fa -w 16
Running time: 5m53.680s

3. Created subsets of all k-mers that occur at least 10 times in sex-chromsome lists
glistcompare chrX.fa_16.list chrX.fa_16.list -i -c 10 -o X_ge10
Running time: 0m1.596s
NUnique 311364
NTotal 17923151
glistcompare chrY.fa_16.list chrY.fa_16.list -i -c 10 -o Y_ge10
Running time: 0m0.155s
NUnique 419403
NTotal 33884614

4. Subtracted the autosome list from repeated k-mer lists using cutoff 2
glistcompare X_ge10_16_intrsec.list autosomes_16.list -d -c 2 -o X_ge10_A_lt2
Running time: 0m12.151s
NUnique 2629
NTotal 17638
glistcompare Y_ge10_16_intrsec.list autosomes_16.list -d -c 2 -o Y_ge10_A_lt2
Running time: 0m12.505s
NUnique 117479
NTotal 3756977

5. Subtracted other sex-chromosme list from repeated and unique k-mer lists using cutoff 1
glistcompare X_ge10_A_lt2_16_0_diff1.list chrY.fa_16.list -d -c 1 -o X_ge10_A_lt2_Y_lt1
Running time: 0m0.102s
NUnique 2461
NTotal 37970
glistcompare Y_ge10_A_lt2_16_0_diff1.list chrX.fa_16.list -d -c 1 -o Y_ge10_A_lt2_X_lt1
Running time: 0m1.023s

NUnique 112387
NTotal 6998558

As there were 112387 Y-specific k-mers we further removed those that had more than 50 copies to get rid of well-known repeats.

6. Created subset of all k-mers that occur at least 50 times Y list
glistcompare Y_ge10_16_intrsec.list Y_ge10_16_intrsec.list -i -c 50 -o Y_ge50
Running time: undetectable
NUnique 259986
NTotal 29972854

7. Subtracted the list of k-mers of over 50 copies from the list of Y-specific k-mers
glistcompare Y_ge10_A_lt2_X_lt1_16_0_diff1.list Y_ge50_16_intrsec.list -d -c 1 -o Y_ge10_lt50_A_lt2_X_lt1
Running time: undetectable
NUnique 49950
NTotal 1183903

We got in total 2461 X-specific and 49950 Y-specific k-mers.

The next step in analysis was finding regions in chromosome which had significant overrepresentation of unique repeated k-mers, using custom Perl script find_regions.pl.
Finding MA > 4, length 100 regions
Y: 2080 regions
X: 88 regions

Blast regions against themselves to locate similarity groups. Collated groups and chose representative sequence.
Scripts:
collate_repeats.pl
filter_collated.pl
unique.pl
Finally we did new BLAST search of identified repeat sequences against the whole genome and filtered truly unique repeats
filter_final.pl

In total we found 319 repeats (11 for X and 308 for Y chromosome).
